# Two-dimensional array of iron-garnet nanocylinders supporting localized and lattice modes for the broadband boosted magneto-optics

**DOI:** 10.1515/nanoph-2021-0534

**Published:** 2021-11-04

**Authors:** Polina E. Zimnyakova, Daria O. Ignatyeva, Dolendra Karki, Andrey A. Voronov, Alexander N. Shaposhnikov, Vladimir N. Berzhansky, Miguel Levy, Vladimir I. Belotelov

**Affiliations:** Moscow Institute of Physics and Technology, National Research University, Dolgoprudny, Moscow 141701, Russia; Russian Quantum Center, Moscow, Russia; Faculty of Physics, M.V. Lomonosov Moscow State University, Moscow, Russia; V.I. Vernadsky Crimean Federal University, Simferopol, Russia; Physics Department, Michigan Technological University, Houghton, USA

**Keywords:** all-dielectric nanostructures, enhancement of magneto-optical effects, Faraday effect, transverse magneto-optical Kerr effect

## Abstract

We demonstrate a novel all-dielectric magnetophotonic structure that consists of two-dimensional arrays of bismuth substituted iron-garnet nanocylinders supporting both localized (Fabry–Perot-like) and lattice (guided-like) optical modes. Simultaneous excitation of the two kinds of modes provides a significant enhancement of the Faraday effect by 3 times and transverse magneto-optical Kerr effect by an order of magnitude compared to the smooth magnetic film of the same effective thickness. Both magneto-optical effects are boosted in wide spectral and angular ranges making the nanocylinder array magnetic dielectric structures promising for applications with short and tightly focused laser pulses.

## 1 Introduction

Nowadays, the magneto-optical effects are widely used in different devices [1] such as routers [2], optical isolators [3–5], magneto-optical sensors [6–12], modulators, and magnetometers [13–16]. From the point of view of the device miniaturization, it is important to design the nanostructures with efficient magneto-optical interaction providing the enhancement of the magneto-optical effects compared to the same smooth films.

First, we focused on the enhancement of the magneto-optical Faraday effect which is the rotation of linear polarization of light passing through a material along an external magnetic field. The value of the rotation angle is directly proportional to the specific Faraday rotation of the material and the path traversed by light in the material. Since there is a dependence on the length of the path, miniaturization of devices inevitably leads to a decrease in the Faraday rotation value. Therefore, it is important to find ways to improve the magneto-optical response of the materials. One of the straightforward ways to enhance the magneto-optical Faraday rotation in thin films is the utilization of the photonic crystals with microcavity (defect) magnetic layers [7, 17], [18], [19], [20], [21], [22], [23], [24], [25], [26]. Photonic crystals surrounding a magnetic layer act as a Bragg mirrors leading to the multiple rereflection of light inside the magnetic layer like in the Fabry–Pérot cavity. Faraday polarization rotation constantly increases each loop the light travels inside the magnetic layer. Such amplification of the Faraday effect can be attributed to the increase in the effective path length of light through the material.

At the same time, it is possible to enhance magneto-optical effects via excitation of the optical resonances in the magnetic nanostructures, which can be attributed to the increase in the interaction time between light and a magnetic medium. Various types of nanostructures were shown to increase the magneto-optical effects [27, 28]. For example magneto-plasmonic crystals offer an opportunity to increase the Faraday and Kerr effects due to excitation of surface plasmon polaritons [29–37]. The enhancement could be observed due to the excitation of plasmonic resonances in metallic particles or nanoantennas [38–44], plasmonic nanocavities [45] and in the artificial metal-dielectric composites with hyperbolic dispersion [46]. The possibility of the enhancement of the magneto-optical effects was also reported for structures that maintain simultaneously several kinds of optical modes [47, 48]. However, metals in such structures cause high absorption losses, broadening of observed resonances and decrease of the base (transmitted) signal.

This problem can be solved by using all-dielectric resonant nanostructures [49–53]. All-dielectric structures are characterized by nearly-zero absorption, so their transmittance and reflectance could be tuned in a wide range by the structure design. Recent studies [51, 53] show that one may obtain high magneto-optical intensity modulation in the transverse configuration of the external magnetic field applied to the structure. However, such amplification is observed in a very small angular and wavelength range due to the high *Q*-factor of the guided wave resonances. On the contrary, Mie resonances in all-dielectric structure possess very low *Q*-factors and provide rather low magneto-optical effects enhancement [54].

Here we show the enhancement of the Faraday and transverse magneto-optical Kerr effect (TMOKE) in the two-dimensional arrays of cylinders made of bismuth-substituted iron-garnet that support both localized (Fabry–Pérot-like) and lattice (guided-like) modes. Simultaneous excitation of these modes makes it possible to increase the Faraday rotation by 3 times compared to the smooth magnetic film of the equal effective thickness. The one-order increase of TMOKE is also observed in the structure in a wide angular range.

## 2 Optical modes of the iron-garnet nanocylinder 2D array

**Figure 1:**
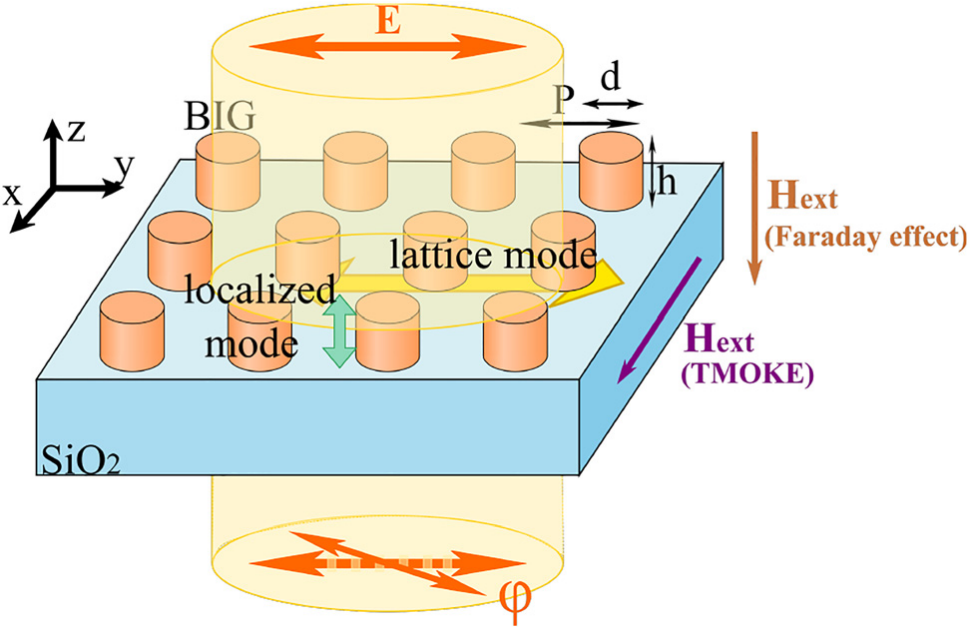
Schematic representation of the BIG nanocylinder 2D array and the excited localized and lattice modes.

The samples under research are two-dimensional arrays of cylinders etched in bismuth-substituted iron garnet (BIG) thin film (the thickness is 515 nm) of Bi_1.0_Lu_0.5_Gd_1.5_Fe_4.2_Al_0.8_O_12_/Bi_2.8_Y_0.2_Fe_5.0_O_12_ deposited by magnetron sputtering on a SiO_2_ substrate (Figure 1). The nanocylinders were patterned on a 550 nm thick spin-coated positive e-beam resist (ZEP-520A) by electron-beam exposure with a uniform dose of 140 μC/cm^2^ and under proximity effect correction (PEC) using a 100 keV e-beam lithography system (VISTEC EBPG 5000+). A 30 nm-thick gold layer was also coated on top to avoid electrical charging of the dielectric garnet film during e-beam exposure. After which, the gold layer was first removed by wet etching in a gold etchant solution and then the resist was developed in an amyl acetate solution. The resist patterns were then transferred onto the BIG film by sputter-etching at a rate of 2.5 nm/min with an argon-ion beam. The temperature of the sample stage was maintained at 60 °C throughout the etching process to avoid hardening of the resist, which was then removed using resist remover *N*-methyl-2-pyrrolidine (NMP) by heating at 800 °C for about half an hour. The BIG cylinders with diameter *d* (*d* = 500 nm, 550 nm, 600 nm, 650 nm for the studied samples) having the same height *h* = 515 nm were arranged in a square lattice with a period of *P* = 900 nm.

Such nanostructured material supports two kinds of optical modes: the localized and lattice modes. The former could be understood in a frame of the waveguide theory as the modes of cylinder waveguides [55, 56]. The waveguide with a circular cross-section supports various types of modes: TM and TE modes with axially-symmetric polarizations, and hybrid EH modes with the mixed polarization. As in the present experiments, the nanocylinders are illuminated by a rather wide linearly-polarized collimated light beam of ∼300μm diameter with a uniform distribution of the **E** vector at the scale of the nanocylinder, only modes with azimuth independent **E**-field distribution could be excited. Actually, among waveguide modes, only EH_1,*m*_ modes (which correspond to the linearly polarized LP_0,*m*_ modes which are given by the characteristic equation *u*[*J*_*m*−1_(*u*)/*J*_*m*_(*u*)] = −*w*[*K*_*m*−1_(*w*)/*K*_*m*_(*w*)] in the case of weak refractive index contrast [57]) have zero orbital number and azimuth independent spatial distribution of polarization vector inside the core. The field of such mode in the BIG cylinder has the form [55, 56]: *E*_*x*_ = *J*_0_(2*U*_*m*_ *r*/*d*)**e**_**x**_ where **e**_**j**_ is the unit polarization vector, *J*_0_ is a Bessel function, $r=\sqrt{x^{2}+y^{2}}$ is the radial coordinate and $U_{m}=\frac{1}{2}k_{0}d\sqrt{n_{BIG}^{2}-n_{m}^{2}}$ is the dimensionless constant that depends on the mode refractive index *n*_*m*_, *k*_0_ is the wave vector of the incident light.

**Figure 2:**
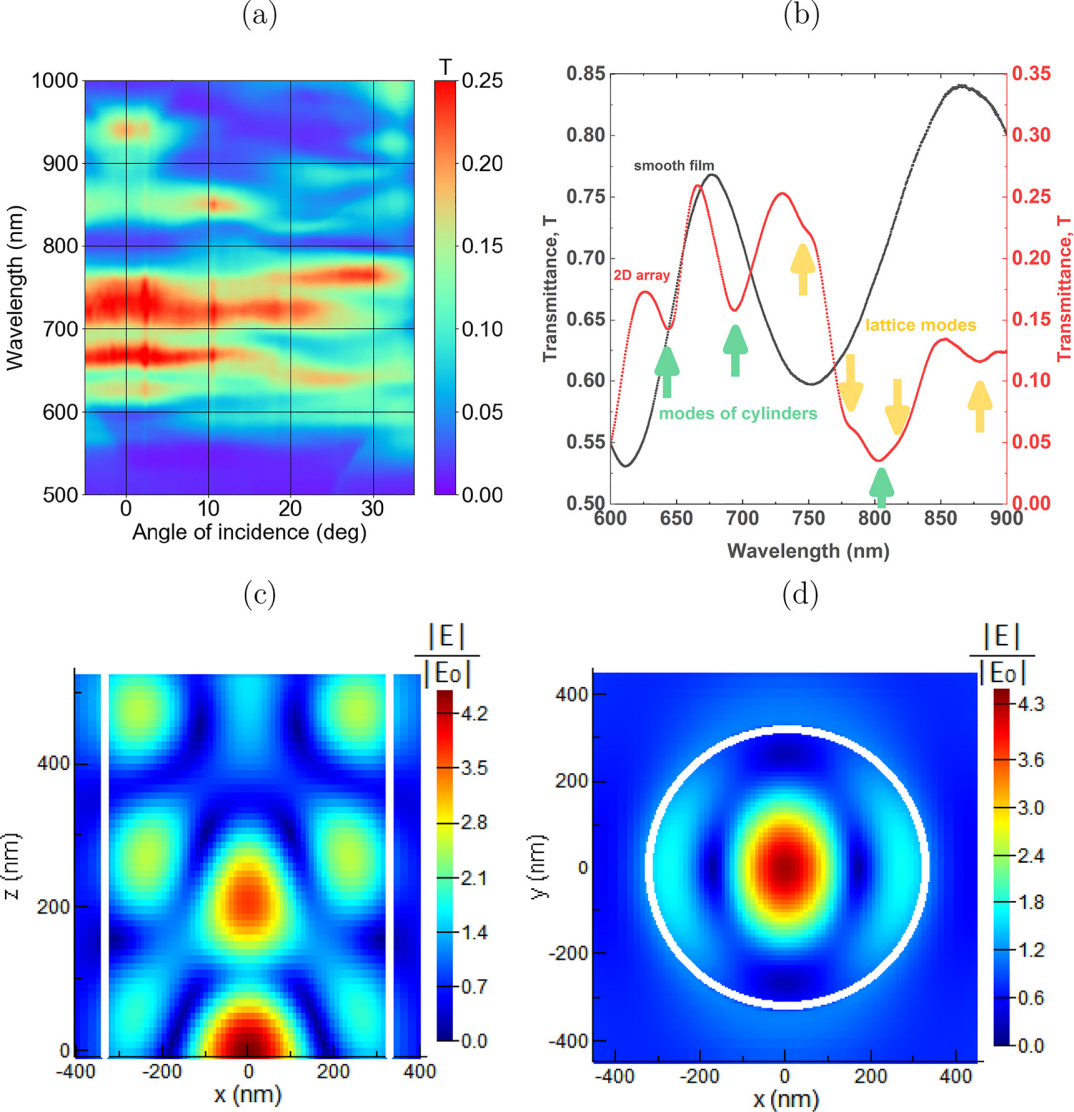
Near- and far-field optical properties of 2D nanocylinder array. (a) False-color plot for the experimental dependence of the transmittance on the light incidence angle and wavelength for the structure with *d* = 650 nm; (b) experimental transmittance spectra of the smooth film (black) and the array of cylinders (red) with *d* = 650 nm (the green (yellow) arrows indicate the dips corresponding to the excitation of the (lattice modes) eigenmodes). (c and d) False-color plots for the spatial distributions of light electric field value |E| at the excitation of the localized mode at *λ* = 800 nm in (c) *x* − *z* and (d) *x* − *y* planes.

We deal with a piece of the cylinder waveguide with the two facets neighbored by air (*n*_a_ = 1) and fused silica ( $n_{{SiO}_{2}}=1.45$ ), correspondingly. These facets act as the mirrors forming a ‘vertical’ Fabry–Pérot cavity (Figure 1) and causing the minima and maxima in the transmittance spectra (see green arrows in Figure 2b). The difference of the mode refractive indices *n*_*m*_ for the modes of different orders leads to the differences in the resonant wavelengths. For example, as the numerical simulations show, the localized mode at *λ* ∼ 800 nm is the EH_1,2_ mode (Figure 2d) forming a standing wave (Figure 2c) inside the BIG cylinder. Excitation of such mode results in the 20-times enhancement of the electromagnetic field intensity inside the cylinder. Numerical simulations were conducted in the Lumerical FDTD Solution software. The simulated object has been defined as a cylinder (the permittivity model has been described elsewhere [53]) placed atop of a semi-infinite glass substrate (refractive index is the same for all wavelengths and equals 1.45). Periodic boundary conditions have been selected along the *x* and *y* axes, PML boundary conditions – along the *z*-axis. The sample was illuminated by a linearly polarized plane wave source along the *z*-axis. The field at Figure 2c and d is normalized to the field of the light source.

The *Q*-factor of such a cavity is rather low; therefore the observed resonances are spectrally rather wide, 50–100 nm in width, approximately. The positions of the observed resonances differ from the interference minima and maxima of the smooth BIG film (Figure 2b) and are independent of the angle of incidence. These localized resonance spectral positions strongly depend on the diameter of the nanocylinder and experience a redshift if the diameter increases.

Figure 2a shows that the angle-dependent lattice modes are also excited in the structure. These modes are formed due to the interaction of the leaking near-field radiation of the localized modes in the cylinders arranged periodically [58–62]. Such modes can be treated as the guided modes propagating in the lateral direction in an effective planar waveguide with a core formed by the nanopatterned BIG film [51–53]. In a sense, these modes are similar to the lattice surface plasmons [63]. In the 2D lattice, such guided modes can be excited under the following phase-matching condition:

(1) $$k_{\tau }+G_{x}l_{x}+G_{y}l_{y}=\beta$$

where **k**_*τ*_ = *k*_0_ sin *θ* **e**_*τ*_ is the tangential component of the incident light wavevector, *θ* is the angle of incidence, *l*_*x*_ and *l*_*y*_ are integers which correspond to the order of the lattice mode, $G_{x}=G_{y}=\frac{2\pi }{P}$ are the absolute values of the reciprocal lattice vectors, *P* is the period of the structure, *λ* is the wavelength of the incidence light, ***β*** is the wave vector of the lattice mode. As the period of the gratings is rather high (*P* > *λ*, $P>n_{{SiO}_{2}}\lambda$ ) and there are several propagating diffraction orders generated in reflection and transmission, the efficiency of the lattice mode excitation is not very high. In Figure 2a these modes reveal themselves intersecting the angle-independent resonances of the localized modes. In Figure 2b lattice modes can be located as bents in the transmittance curve and are pointed with yellow arrows.

As it will be shown below, both of these modes are involved in the magneto-optical interaction of light with the 2D nanocylinder array. We will focus on the analysis of the localized mode EH_1,2_ excited at *λ* ∼ 800 nm as it exists in all of the fabricated structures that make it possible to track the evolution of the magneto-optical response with the variation of the nanocylinder diameter and appearance of the lattice modes in its vicinity.

## 3 The Faraday effect with localized and lattice modes

The magnetic film produces the magneto-optical Faraday rotation of the polarization of the transmitted light under the application of the external magnetic field parallel to the light wavevector. Such magnetization also influences the characteristics of the lattice and localized modes, in different ways.

The localized modes of the nanocylinders have polarization degeneracy in the nonmagnetic case, i.e. the two EH_1,*m*_ modes of the same order *m* and orthogonal polarizations **e**_*x*_ and **e**_*y*_ have the same refractive index *n*_1,*m*_ [56]. Modes with circular polarization unit vectors **e**_*x*_ + *i* **e**_*y*_ and **e**_*x*_ − *i* **e**_*y*_ are the eigenmodes of the system with the same refractive index *n*_1,*m*_. Similar to the case of a smooth film, application of the external magnetic field along the cylindrical waveguide axes lifts this degeneracy so that the refractive indices of both modes acquire additional magneto-optical terms of different signs. This birefringence between the two circularly polarized modes excited by the linearly-polarized incident light results in the Faraday rotation of its polarization. Notice that due to the complex dispersion of the nanocylinder modes such magneto-optical circular birefringence differs from the one in a smooth film.

The lattice modes are also sensitive to the magnetization; however, the situation is more complicated. These modes have linear eigenpolarization in the nonmagnetic structure (for example, the TM-mode with {*E*_*x*_, *E*_*z*_, *H*_*y*_}). An external magnetic field applied normally to the structure changes these exact analytical solutions of Maxwell’s equations and gives the different polarization of the eigenmodes. The eigenmode still has the same ({*E*_*x*_, *E*_*z*_, *H*_*y*_} for TM, for example) components but acquires linear in magnetization orthogonal (TE in the considered case {*E*_*y*_, *H*_*x*_, *H*_*z*_}) components [64]. The additional components are not associated with some other mode of the orthogonal polarization existing in the structure and appear just as a rigorous solution of Maxwell’s equations for the corresponding constitutive equations.

It is important that the presence of the interface of the materials and the corresponding boundary conditions for the electromagnetic field also impose restrictions on the Faraday-like rotation of the polarization during the propagation along the surface [65]. Thus quasi-TM and quasi-TE modes appear in the magnetized medium. The refractive indices of the quasi-TM and quasi-TE modes remain the same as for the non-magnetic case. However, for rather thick magnetic films the dispersions of the quasi-TM and quasi-TE modes are very close to each other so that the appearance of the magneto-optically induced orthogonal polarization components results in the energy swap between the quasi-TM and quasi-TE resulting in the polarization rotation [66]. This mechanism is less efficient as the lattice modes are rather shallow due to the high scattering during the propagation in a lateral direction. Nevertheless, both types of the modes and both mechanisms are involved in the Faraday rotation observed in the structure as discussed below.

To compare the Faraday rotations in both cases correctly, we have to take into account the fact that the 2D arrays with different diameters of the nanocylinders have different specific amounts of the magnetic BIG material per period. This amount is also different from the one in the smooth film of the same physical thickness. On the other hand, the specific Faraday rotation itself depends on the wavelength of the light thus it will inevitably differ for the resonances excited at different wavelengths. Therefore, to compare the nanostructure-induced enhancement of the Faraday rotation angles obtained in various structures more correctly, we calculate the value of relative enhancement as:

(2) $$\phi \left(\lambda \right)=\frac{4P^{2}}{\pi d^{2}}\frac{\Phi_{arr}\left(\lambda \right)}{\Phi_{\mathrm{fi}lm}\left(\lambda \right)}$$

where Φ_arr_ is the Faraday rotation angle measured in a 2D array of cylinders, Φ_film_ is the Faraday rotation angle measured in the smooth BIG film of the same thickness and composition, and the factor $\frac{4P^{2}}{\pi d^{2}}$ accounts for the variation of the relative amount of BIG material.

**Figure 3:**
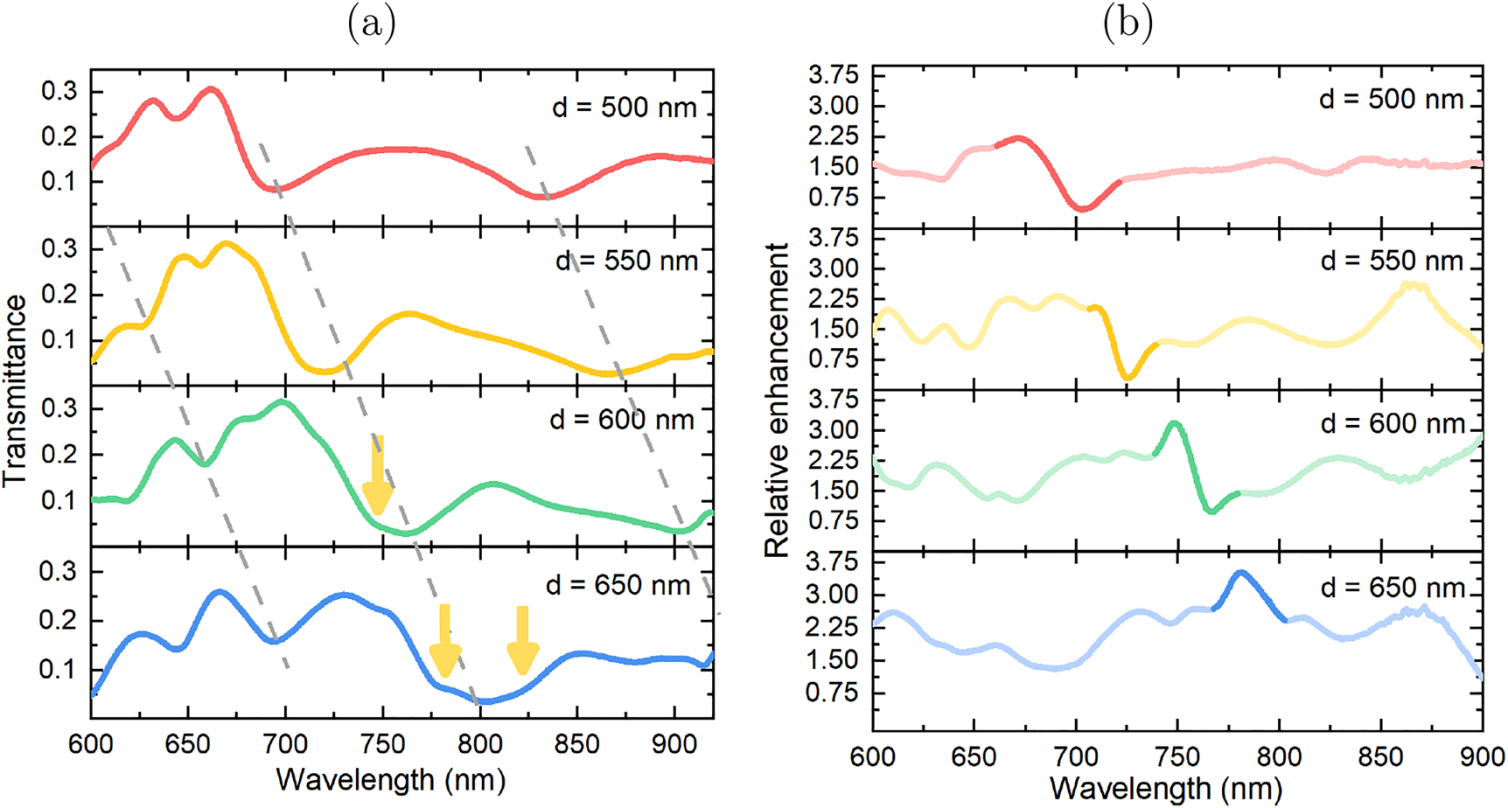
The transmittance (a) and the relative enhancement of the Faraday rotation (b) in the nanocylinder arrays with different nanocylinder diameters (see the legends). In (b) the spectral range in the vicinity of EH_1,2_ localized resonance is highlighted.

Figure 3a shows that for structures with diameters of cylinders *d* = 500 nm and *d* = 550 nm at the wavelengths in the vicinity of 700 nm, only the localized eigenmode of cylinders is excited. The relative enhancement of the Faraday effect in these structures is less than 2 times in comparison with a smooth BIG film. For structure with *d* = 600 nm in the vicinity of the localized eigenmode excitation (*λ* = 770 nm) the lattice mode is also excited so that the *ϕ* spectra acquire the Fano shape due to the superposition of these two resonances. Simultaneous excitation of the same eigenmode and two lattice modes in the structure with *d* = 650 nm results in the interaction of the three magneto-optical resonances. This leads to the further change of the resonance shape which is more significant and makes the relative enhancement value larger up to 3.75 times in comparison with that of a smooth iron garnet film.

Therefore, the simultaneous excitation of the localized and lattice modes is shown to be responsible for nearly 4-times relative enhancement in Faraday rotation concerning a smooth film of the same thickness. Due to the same configuration of the external magnetic field for the polar magneto-optical Kerr effect and the Faraday effect one could expect a similar enhancement of PMOKE.

## 4 Transverse magneto-optical Kerr effect with localized and lattice modes

The transverse magnetic field applied to the structure causes the magneto-optical Kerr effect that results in the modulation of the transmitted or reflected light intensity. This effect is below 10^−4^ in magnitude for the smooth iron-garnet films; however, it can be enhanced by the mode excitation.

The mechanism of the TMOKE amplification under the excitation of the lattice modes is the nonreciprocal magneto-optical variation of the mode propagation constant *β* = *β*(*H* = 0) + Δ*β*(*H*) that changes the resonance conditions according to Eq. (1) and causes a variation of the intensity of the transmitted and reflected light. It is important that cylinder bases in our structures are surrounded with different materials with different refractive indexes (air and SiO_2_, correspondingly). If the structure symmetry would be spatially symmetric with respect to the *z*-axis, the TMOKE would be prohibited. A detailed description of this mechanism for all-dielectric gratings was presented elsewhere [51]. Although it allows one to observe a strong enhancement of TMOKE, this enhancement is provided in a very narrow angular and frequency range in the vicinity of the lattice mode resonances. This limitation could be overcome if localized modes with angular-independent and wide resonances are excited simultaneously with the lattice ones.

**Figure 4:**
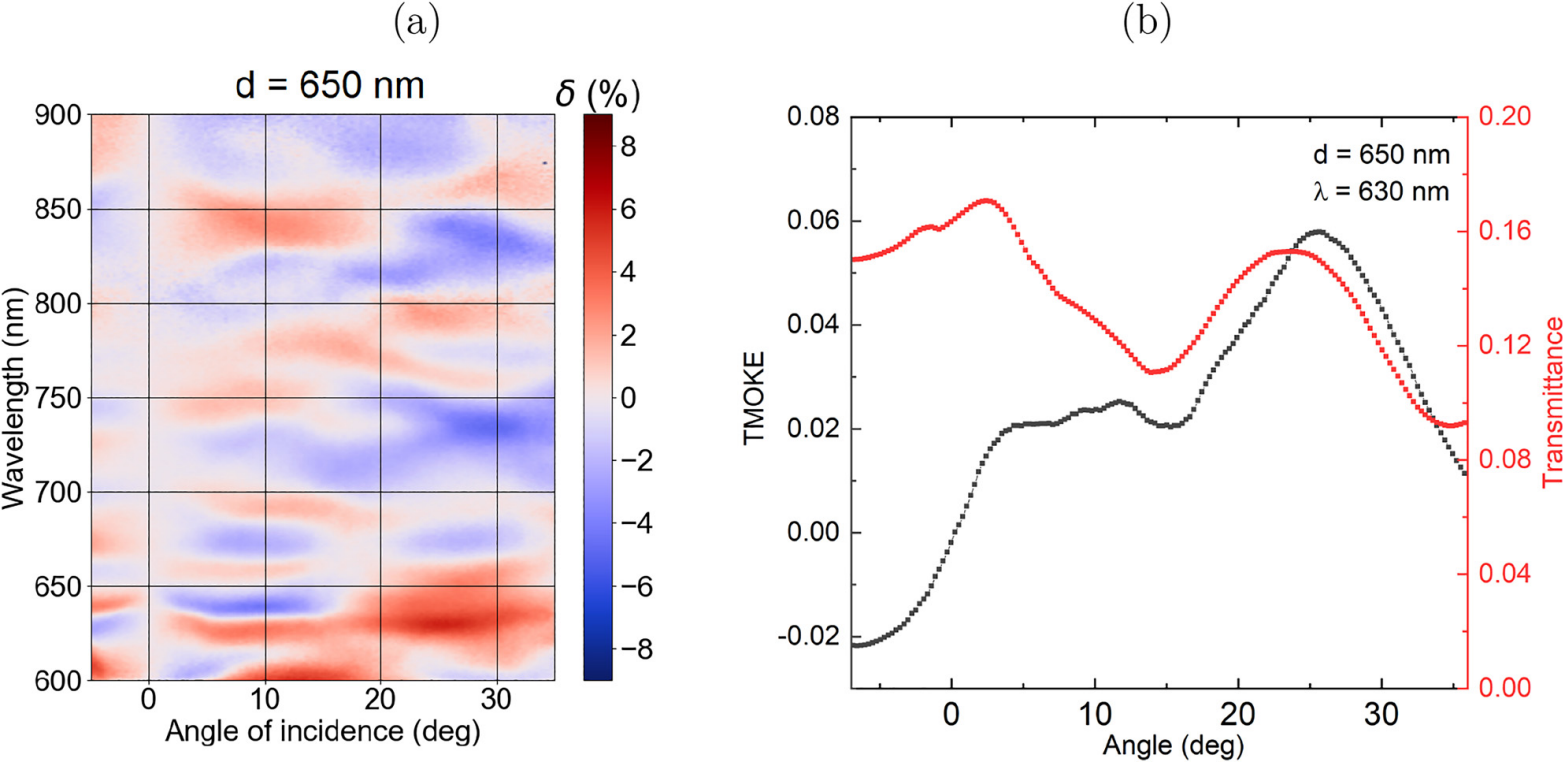
TMOKE in transmission for nanocylinder array with *d* = 650 nm. (a) False-color plot of the dependence of TMOKE of the light with the incident angle and the wavelength. (b) Angular dependence of TMOKE and transmittance at the wavelength *λ* = 630 nm.

Figure 4 shows the TMOKE spectra *δ* for the nanocylinder array with *d* = 650 nm. Measurements were conducted for transmitted light and the results shown in Figure 4a are defined as

(3) $$\delta =\frac{T\left(+M\right)-T\left(-M\right)}{\frac{1}{2}\left(T\left(+M\right)+T\left(-M\right)\right)}\cdot 100\%,$$

where *T* is the transmittance of an array and **M** is the magnetization of the sample. One may see that the TMOKE is enhanced in the whole measurement range and the resonances are broad and have large angular width. The most interesting is the TMOKE enhancement up to 5% for *λ* = 630 nm and exceeds 2% for the angles of incidence from 5 to 35°. It is accompanied by the rather high transmittance value of *T* = 16%. It opens up new possibilities for the device miniaturization since such wide spectra allow one for the utilization of tightly focused light for efficient magneto-optical modulation.

## 5 Conclusions

All-dielectric structure possess near-zero ohmic losses. The transparency of such structure depends only on its geometrical configuration and can be tuned in a wide range. In our experiments, the transmittance was about 16%, which allowed us to observe the magneto-optical polarization and intensity effects in the transmitted light, while usually studies of the plasmonic structures focus on the reflectance configuration [28, 42], [43], [44]. We show that such transparency is accompanied by 20-times enhancement of the light intensity inside the magnetic material of the structure and significant amplification of the magneto-optical effects.

The enhancement of the Faraday and transverse magneto-optical Kerr effects in the two-dimensional arrays of nanocylinders made of bismuth-substituted iron-garnet were shown experimentally. The feature of the considered structure is the coexistence of both localized (Fabry–Pérot-like) and lattice (guided-like) modes, which are both responsible for the enhancement of the magneto-optical response. The Faraday effect depends on the effective path that is passed by light in the magnetic medium. It is increased by the Fabry–Pérot resonances attributed to the individual cylinders. Excitation of the lattice resonances corresponds to the ‘synchronisation’ of these individual particles and thus provides further Faraday effect amplification. TMOKE, in its turn, usually arises when the structure possesses a spatial non-reciprocity. In our case, it is the *k*-vector of the lattice modes that differs for the two opposite directions of the magnetic field applied to the structure. Excitation of the additional localized Fabry–Pérot resonances in each cylinder of the lattice leads to the increase of the efficiency of the interaction of light with a lattice and thus further enhances TMOKE. Simultaneous excitation of these modes makes it possible to increase the Faraday rotation by 3 times compared to that in the smooth magnetic film of the equal effective thickness. The one order of magnitude increase in TMOKE is also observed in the structure. It is important that both the Faraday rotation and TMOKE are enhanced in wide spectral and angular ranges. This makes the considered structure prospective for magneto-optical applications with tightly focused femtosecond laser pulses that usually have rather broad frequency and angular spectra.
